# Augmentation of tumor expression of HLA-DR, CXCL9, and CXCL10 may improve olfactory neuroblastoma immunotherapeutic responses

**DOI:** 10.1186/s12967-024-05339-9

**Published:** 2024-05-31

**Authors:** Riley M. Larkin, Diana C. Lopez, Yvette L. Robbins, Wiem Lassoued, Kenneth Canubas, Andrew Warner, Baktiar Karim, Ksenia Vulikh, James W. Hodge, Charalampos S. Floudas, James L. Gulley, Gary L. Gallia, Clint T. Allen, Nyall R. London

**Affiliations:** 1 Sinonasal and Skull Base Tumor Program, Surgical Oncology Program, Center for Cancer Research, National Cancer Institute, National Institutes of Health, Bethesda, MD USA; 2 University of Miami Miller School of Medicine, Miami, FL USA; 3 Department of Otolaryngology-Head and Neck Surgery, Johns Hopkins University School of Medicine, Baltimore, MD USA; 4 Section on Translational Tumor Immunology, Center for Cancer Research, National Cancer Institute, National Institutes of Health, Bethesda, MD USA; 5 Center for Immuno-Oncology, Center for Cancer Research, National Cancer Institute, National Institutes of Health, Bethesda, MD USA; 6 Molecular Histopathology Laboratory, Frederick National Laboratory for Cancer Research, Frederick, MD USA; 7 Department of Neurosurgery, Johns Hopkins University School of Medicine, Baltimore, MD USA

**Keywords:** Esthesioneuroblastoma, Immunotherapy, Olfactory neuroblastoma

## Abstract

**Background:**

Olfactory neuroblastoma is a rare malignancy of the anterior skull base typically treated with surgery and adjuvant radiation. Although outcomes are fair for low-grade disease, patients with high-grade, recurrent, or metastatic disease oftentimes respond poorly to standard treatment methods. We hypothesized that an in-depth evaluation of the olfactory neuroblastoma tumor immune microenvironment would identify mechanisms of immune evasion in high-grade olfactory neuroblastoma as well as rational targetable mechanisms for future translational immunotherapeutic approaches.

**Methods:**

Multispectral immunofluorescence and RNAScope evaluation of the tumor immune microenvironment was performed on forty-seven clinically annotated olfactory neuroblastoma samples. A retrospective chart review was performed and clinical correlations assessed.

**Results:**

A significant T cell infiltration was noted in olfactory neuroblastoma samples with a stromal predilection, presence of myeloid-derived suppressor cells, and sparse natural killer cells. A striking decrease was observed in MHC-I expression in high-grade olfactory neuroblastoma compared to low-grade disease, representing a mechanism of immune evasion in high-grade disease. Mechanistically, the immune effector stromal predilection appears driven by low tumor cell MHC class II (HLA-DR), CXCL9, and CXCL10 expression as those tumors with increased tumor cell expression of each of these mediators correlated with significant increases in T cell infiltration.

**Conclusion:**

These data suggest that immunotherapeutic strategies that augment tumor cell expression of MHC class II, CXCL9, and CXCL10 may improve parenchymal trafficking of immune effector cells in olfactory neuroblastoma and augment immunotherapeutic responses.

**Supplementary Information:**

The online version contains supplementary material available at 10.1186/s12967-024-05339-9.

## Background

Olfactory Neuroblastoma (ONB), also known as esthesioneuroblastoma, is a very rare malignancy of the nasal cavity and anterior skull base. The incidence is 4 per 10 million persons per year and accounts for 2–3% of all malignant sinonasal tumors [1, 2]. Although outcomes are fair for local disease there remains a cohort of patients with high-grade, recurrent, or metastatic disease who do not respond well to standard therapy necessitating development of additional treatment options [3–5]. Indeed, prognosis for high-grade disease is poor with a 5- and 10-year overall survival of 60.9% and 49.6% respectively [3]. When amenable, treatment typically consists of surgery followed by adjuvant radiation [6]. Adjuvant or neoadjuvant chemotherapy is often reserved for locoregionally advanced or metastatic disease [6–8]. There are no approved targeted therapies for ONB, and current treatment approaches can lead to significant long-term morbidity [9]. The role of immunotherapy has not been extensively evaluated in ONB, with only one currently available ONB-specific interventional trial available in the United States (NCT05012098); investigating the bifunctional fusion protein Bintrafusp alfa, which targets programmed cell death-ligand 1 (PD-L1) and transforming growth factor-beta (TGF-β) signaling.

The tumor microenvironment consists of a bidirectional communication between a tumor and its associated stroma, which directly influences disease initiation and tumor progression. The stroma is comprised of immune cells, blood vessels, nerves, fibroblasts, and the extracellular matrix [10, 11]. The tumor immune microenvironment (TIME) is characterized by a diverse array of immune cells which interact with tumor cells. T cells have important and well described roles in tumor cell killing and are the most important target for immunotherapeutic approaches to date [12]. The presence of a subset of myeloid cells, termed myeloid-derived suppressor cells (MDSCs), within the TIME has been associated with immunosuppression and a decreased clinical immunotherapy response [13–15]. Natural killer (NK) cells are viable therapeutic targets known to kill adjacent cells independent of antigen presentation [16]. Cytokines and chemokines are known to drive immune cell localization within the TIME. For example, chemokine (C-X-C motif) ligand 9 and 10, (CXCL9 and CXCL10, respectively), mediate recruitment of T cells along chemokine gradients into tumors [17, 18]. Conversely, interluekin-8 (IL-8) promotes epithelial-to-mesenchymal transition and has been shown to recruit MDSCs including neutrophils into tumors [19, 20].

Previous analyses of ONB’s TIME have been superficial and limited to single marker immunohistochemistry [21–23]. Multispectral immunofluorescence (mxIF) is a technique that allows for simultaneous visualization of six markers of interest and phenotype analysis at the cellular level. When paired with machine learning software this technique allows for objective quantification and spatial analysis at single cell resolution. Herein, we describe three panels to comprehensively define the T cell, myeloid, and NK cell compartments as well as a panel to determine major histocompatibility complex class I and II (MHC-I and MHC-II) expression. We hypothesized that an in-depth evaluation of the ONB TIME would identify mechanisms of immune evasion in high-grade ONB as well as rational targetable mechanisms for future translational immunotherapeutic approaches.

## Results

### Patient population clinical characteristics

Forty-seven clinically annotated samples in triplicate were analyzed in this study, including forty new diagnoses and seven locally recurrent lesions (Table 1). The patients were 59.6% male (*n* = 28) and 40.4% female (*n* = 19), with a median follow up time of 98 months (range 3-555). Seventeen patients (36.2%) were high-grade disease (Hyams grade III or IV). Most patients were either Dulgerov Stage T2 (27.7%, *n* = 13) or T3 (25.5%, *n* = 12) and there was a Kadish Stage C predominance (72.3%, *n* = 34). Patients in this cohort presented with a wide variety of symptoms the most common being nasal obstruction (51.1%, *n* = 24), epistaxis (42.6%, *n* = 20), and anosmia (23.4%, *n* = 11). Dural infiltration was present in 38.3% (*n* = 18) and there was orbital involvement in 6.4% (*n* = 3) of cases. Of the forty-seven patients, 36.2% (*n* = 17) experienced post-treatment recurrence and 14.9% (*n* = 7) succumbed to their cancer.

**Table 1:** Patient demographics

**Age at Diagnosis (years)**	
Median (range)	54 (21–90+)
**Sex, n (%)**	
Male	28 (59.6%)
Female	19 (40.4%)
**Follow-Up (months)**	
Median (range)	98 (3-555)
**Sample Type, n (%)**	
Primary	40 (85.1%)
Recurrent	7 (14.9%)
**Hyams Grade, n (%)**	
I	7 (14.9%)
II	22 (46.8%)
III	12 (25.5%)
IV	5 (10.6%)
Unknown	1 (2.1%)
**Modified Kadish Stage, n (%)**	
A	4 (8.5%)
B	4 (8.5%)
C	34 (72.3%)
D	5 (10.6%)
**Dulgerov Stage Sample, n (%)**	
T1	8 (17.0%)
T2	13 (27.7%)
T3	12 (25.5%)
T4	10 (21.3%)
Unknown	4 (8.5%)
**Presenting Symptoms, n (%)**	
Dural infiltration	18 (38.3%)
Orbital involvement	3 (6.4%)
Nasal obstruction	24 (51.1%)
Rhinorrhea	9 (19.1%)
Epistaxis	20 (42.6%)
Anosmia	11 (23.4%)
Headache	8 (17.0%)
Epiphora	1 (2.1%)
Diplopia	1 (2.1%)
Pain	8 (17.0%)

### T cells are present and predominantly localized in the ONB stroma

**Fig. 1:**
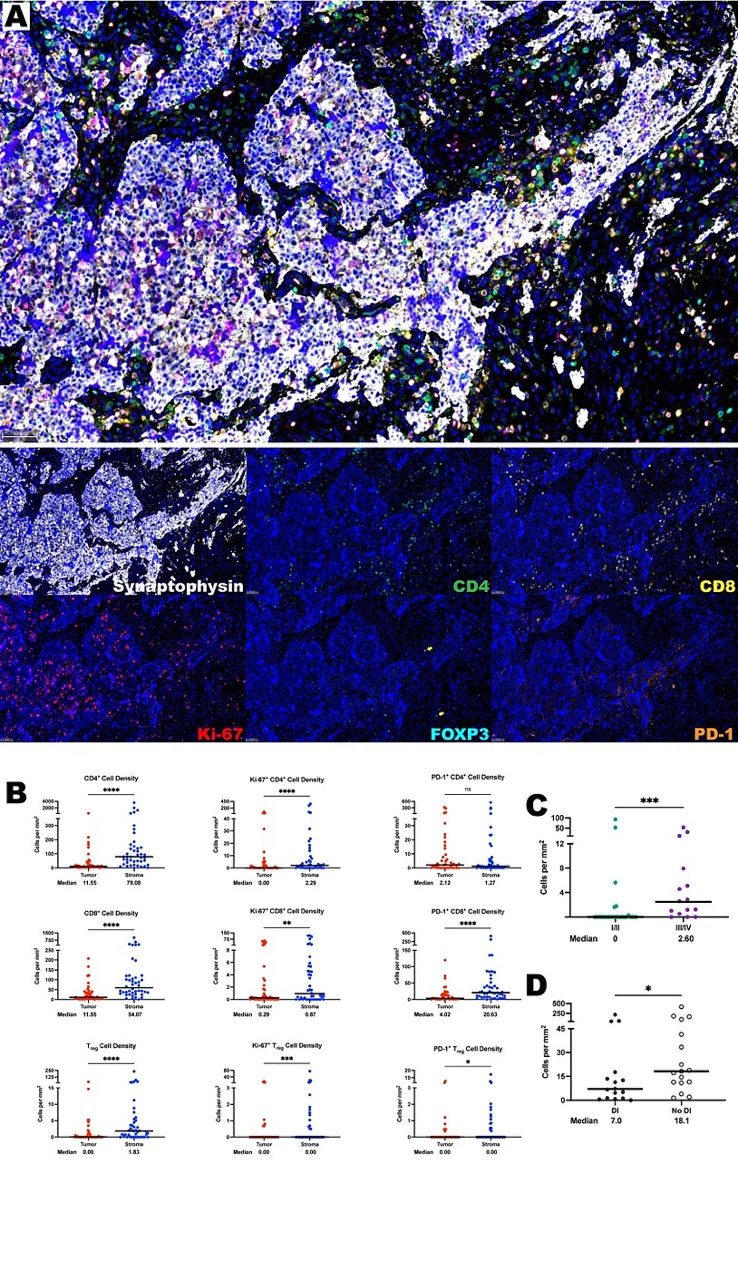
T cells are found in abundance within the olfactory neuroblastoma tumor immune microenvironment, most often within the stroma. **(A)** Representative photomicrographs at 20x magnification from a high Hyams grade ONB of merged and single-color immunofluorescence images assessing the presence of T cells with a validated panel of six biomarkers, CD8 (yellow, Opal 570), CD4 (green, Opal 520), FOXP3 (turquoise, Opal 480) indicated by yellow arrows, Ki67 (red, Opal 690), PD-1 (orange, Opal 620), and synaptophysin (white, Opal 780). Multispectral immunofluorescence images are counterstained with DAPI. Expression of CD4 without CD8 and FOXP3 identified CD4^+^ T helper cells, while expression of CD8 without CD4 and FOXP3 identified CD8^+^ cytotoxic effector T cells. Co-localization of CD4 and FOXP3 without CD8 identified T_regs_. T cells with positive Ki-67 nuclear expression were proliferating, and PD-1 positivity denoted PD-1^+^ T cells. Expression of synaptophysin identified tumor cells. **(B)** Quantification of CD4^+^ T cell, CD8^+^ T cell, and T_reg_ cell density (cells per mm^2^), as well as proliferating and PD-1^+^ subcategories of these are compared between ONB tumor and stroma (*n* = 44 tumor cores). **(C)** Quantification of tumor specific Ki-67^+^ CD4^+^ T helper cells compared by Hyams Grade. **(D)** Quantification of tumor specific CD4^+^ T helper cells when comparing patients who presented with and without dural infiltration. For paired comparisons, Wilcoxon matched-pairs signed rank test and for unpaired comparisons, Mann-Whitney U tests were used to test for statistical significance. All lines are graphed to indicate the median value. *p ≤ 0.05, **p ≤ 0.01, ***p ≤ 0.001, ****p ≤ 0.0001, ns, non-significant

Tumor infiltration of T cells is critical for tumor antigen recognition and immunotherapy response [24]. Utilizing a mxIF panel of CD4, CD8, FOXP3, PD-1, Ki-67, and synaptophysin; T cells were appreciated throughout the tumor microenvironment of ONB (Fig. 1A). CD8^+^ cells in the absence of CD4 or FOXP3 co-localization were defined as cytotoxic T cells, CD4^+^ cells without CD8 or FOXP3 expression denoted T helper cells, and CD4^+^/FOXP3^+^ cells with a lack of CD8 defined regulatory T cells (T_regs_). These phenotypes were found at medians of 33.9 cells per mm^2^, 43.0 cells per mm^2^, and 0.94 cells per mm^2^, respectively, demonstrating the presence of a significant ONB T cell infiltrate. Targeting the PD-1/PD-L1 axis has shown promise in treating melanoma and non-small cell lung cancer [25, 26] and PD-1^+^ cytotoxic T cells, T helper cells, and T_regs_ are present in the ONB TIME at medians of 2.34 cells per mm^2^, 1.58 cells per mm^2^, and 0 cells per mm^2^. Ki-67^+^ proliferating cytotoxic T cells, T helper cells, and T_regs_ were found in the ONB TIME at medians of 1.58 cells per mm^2^, 2.35 cells per mm^2^, and 0 cells per mm^2^. Next, we utilized segmentation software to delineate the compartment localization of these T cell subtypes. When comparing the localization of these T cell subtypes within the tumor parenchyma and stromal compartments, all T lymphocyte phenotypes of interest were found to have a strong predilection for the stroma except PD-1^+^ T helper cells (Fig. 1B).

The TGF-β signaling pathway in cancer has a dual role, as a tumor suppressor it inhibits tumorigenesis by inducing growth arrest and apoptosis and as a tumor promoter it induces tumor cell migration and stimulates epithelial-to-mesenchymal transition [27]. In ONB, increases in TGF-β signaling have been linked with shorter disease-free survival [28]. TGF-β also incites polarization of CD4^+^ cells into regulatory T cells, which directly inhibit cytotoxic T cell mediated anti-tumor responses. In our cohort, spatial analysis demonstrates that T_regs_ are significantly closer to non-proliferating CD8^+^ than proliferating CD8^+^ cells (medians of 31.91 μm vs. 63.74 μm, *P* < 0.0001) suggesting that T_regs_ may inhibit T cell replication in ONB (Supplementary Fig. 1).

T cell density distributions were compared to clinical factors of interest including Kadish and Dulgerov stages, primary versus recurrent samples, presence of dural infiltration, orbital involvement, biological sex, age ≥ 65, post-treatment recurrence, or death due to ONB. Interestingly, an increased number of tumor parenchyma localized proliferating T helper cells (medians of 2.60 vs. 0.00, *P* = 0.0004) (Fig. 1C) were seen in high Hyams grade samples suggesting that higher grade tumors may be more amenable to tumor antigen recognition. Dural infiltration is a predictor of overall survival and disease-free survival in primary ONB [4] and patients who presented without dural infiltration had more T helper cells in the tumor parenchyma than those who presented with dural infiltration (medians of 18.1 cells per mm^2^ versus 7.0 cells per mm^2^; *P* ≤ 0.05) (Fig. 1D), suggesting that T helper cells may help to limit disease progression and dural infiltration. An increased number of T helper cells were also observed in females compared to males (medians of 17.49 cells per mm^2^ vs. 5.21 cells per mm^2^; *P* ≤ 0.05) (Supplementary Fig. 2). A trend emerged with PD-1^+^ T helper and cytotoxic T cells, both of which were found in higher density when comparing the tumor compartments of primary and locally recurrent samples. Primary samples contained more PD-1^+^ CD4^+^ (median 2.68 cells per mm^2^ vs. 0 cells per mm^2^, *P* = 0.048) and PD-1^+^ CD8^+^ cells (median 5.89 cells per mm^2^ vs. 0 cells per mm^2^, *P* = 0.008) than recurrent samples (Supplementary Fig. 3). This may indicate that primary tumors are more susceptible to PD-1 pathway inhibition than recurrent tumors. Finally, as one would expect, in high Hyams grade samples there were higher tumor cell proliferation indices (15.37 vs. 3.36 *P* = 0.0048) (Supplementary Fig. 4). T cell distributions did not correlate with any other major measured clinical factors including survival (Supplementary Tables 1–3).

### Myeloid derived suppressor cells are present and predominantly localized in the ONB stroma

**Fig. 2:**
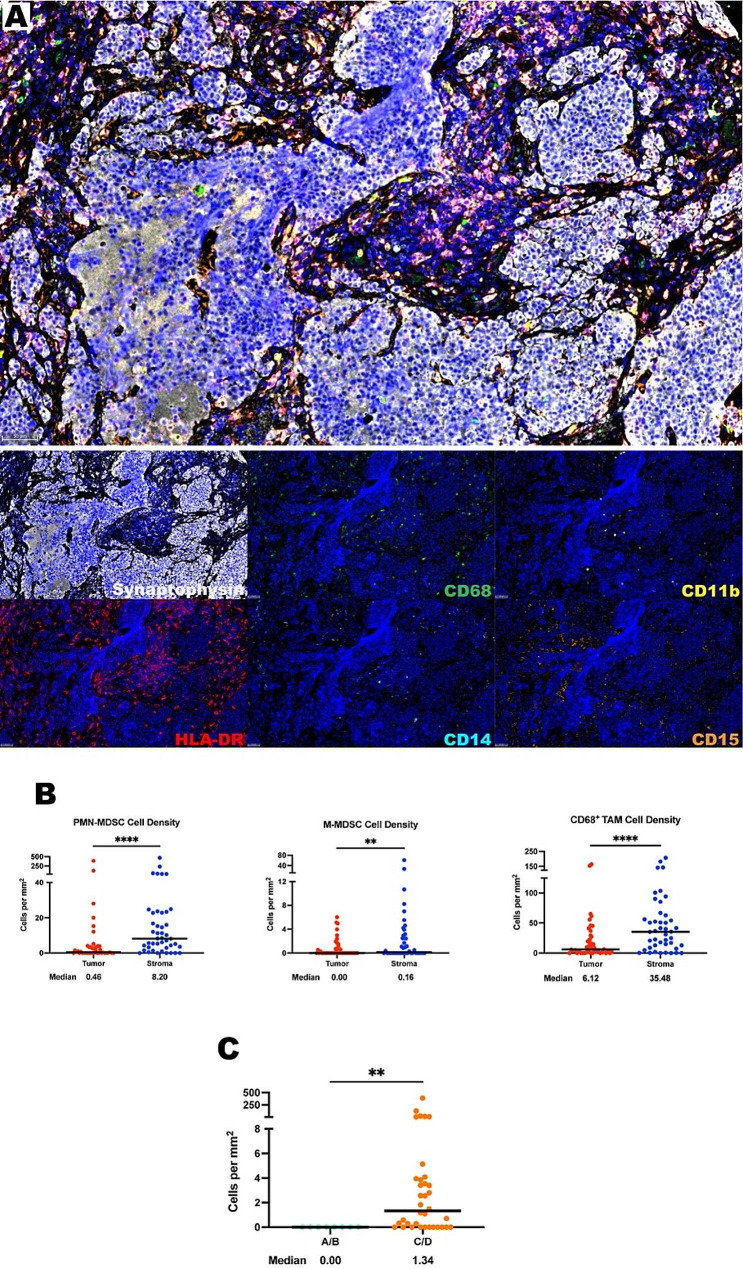
Myeloid cells are found in abundance within the ONB TIME, most often within the stroma. **(A)** Representative photomicrographs at 20x magnification from a low Hyams Grade ONB of merged and single-color immunofluorescence images assessing the presence of myeloid cells with a validated panel of six biomarkers, CD11b (yellow, Opal 570), HLA-DR (red, Opal 690), CD14 (turquoise, Opal 480), CD15 (orange, Opal 620), CD68 (green, Opal 520), and synaptophysin (white, Opal 780). Multispectral immunofluorescence images are counterstained with DAPI. High expression of CD68 identified tumor associated macrophages. Co-localization of CD11b and CD14 without CD15 and little to no HLA-DR identified M-MDSCs. Co-localization of CD11b and CD15 without CD14 and little to no HLA-DR identified PMN-MDSCs. Expression of synaptophysin identified tumor cells. **(B)** Quantification of PMN-MDSCs, M-MDSCs, and CD68^+^ TAMs per mm^2^ in ONB tumor and stroma (*n* = 44 tumor cores in triplicate). **(C)** Tumor PMN-MDSC cell density compared by Kadish stage – Low Kadish Stage (A/B) versus High Kadish Stage (C/D). All lines are graphed to indicate the median value. For paired comparisons, Wilcoxon matched-pairs signed rank test and for unpaired comparisons, Mann-Whitney U tests were used to test for statistical significance. **p ≤ 0.01, ****p ≤ 0.0001

To identify myeloid cells in the ONB TIME a mxIF panel of C11b, HLA-DR, CD14, CD15, CD68, and synaptophysin was used (Fig. 2A). Recent studies have shown that CD68^+^ is overexpressed in tumor associated macrophages (TAMs) [29]. High expressing CD68^+^ TAMs were appreciated at a median density of 19.5 cells per mm^2^, with a higher abundance in the stroma than the tumor (medians of 35.48 cells per mm^2^ vs. 6.12 cells per mm^2^, respectively *P* < 0.0001) (Fig. 2A-B).

The diverse population of myeloid derived suppressor cells can inhibit a plethora of immune effector cells [30]. Two well established groups are monocytic MDSCs (M-MDSCs) which are defined as CD11b^+^/CD14^+^ cells with no CD15 positivity and polymorphonuclear MDSCs (PMN-MDSCs) which are CD11b^+^/CD15^+^ and an absence of CD14, both of which, have little to no expression of HLA-DR. These were found globally at medians of 0.46 cells per mm^2^ and 5.06 cells per mm^2^, respectively. M-MDSCs and PMN-MDSCs are largely excluded from the tumor compartment and restricted to the stroma (Fig. 2A-B).

MDSC immune cell distributions were compared with clinical factors of interest. When comparing Kadish stage, higher stage (C/D) tumors had significantly more tumor infiltrating PMN-MDSCs than low stage (A/B) samples (*P* = 0.0011) (Fig. 2C) suggesting that clinical disease progression may be tied to parenchymal recruitment of this subset of suppressive cells. Although sparse, more M-MDSCs were found in primary samples (medians of 0.49 cells per mm^2^ vs. 0 cells per mm^2^, *P* = 0.0157) (Supplementary Fig. 5). MDSC density distributions did not correlate with any other measured clinical factors including Hyams grade, Dulgerov stages, presence of dural infiltration, biological sex, age ≥ 65, post-treatment recurrence, death due to ONB, or survival. (Supplementary Tables 4–6).

### Natural killer cells are scantly present in the ONB TIME

**Fig. 3:**
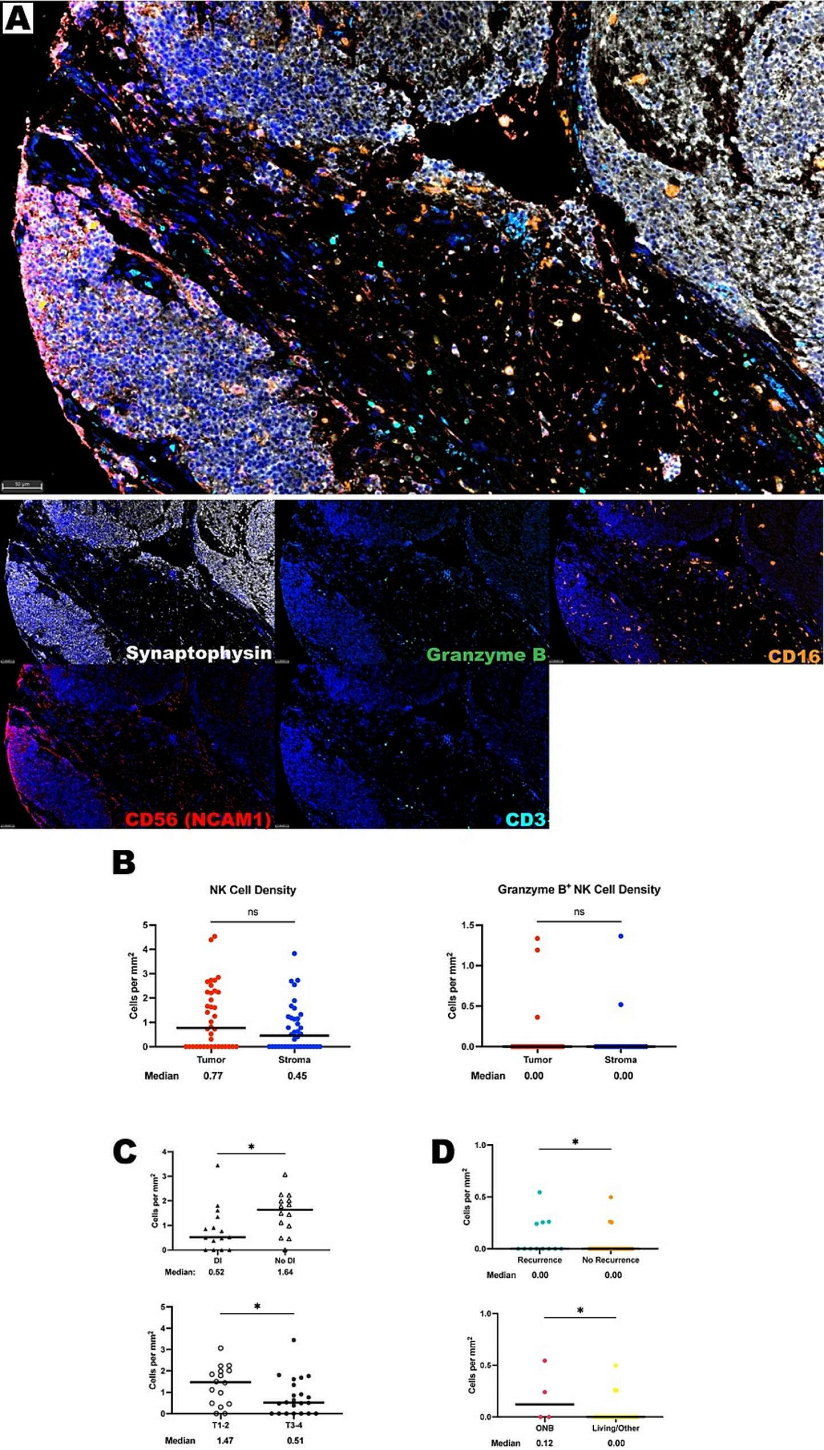
NK cells are sparsely found within the ONB TIME. **(A)** Representative photomicrographs at 20x magnification from a low Hyams Grade ONB of merged and single-color immunofluorescence images assessing the presence of NK cells with a validated panel of five biomarkers, CD16 (orange, Opal 620), CD56 (red, Opal 690), CD3 (turquoise, Opal 480), Granzyme B (green, Opal 520), and synaptophysin (white, Opal 780). Multispectral immunofluorescence images are counterstained with DAPI. Co-localization of CD16 and CD56 without CD3 and a decrease size threshold compared to tumor cells identified NK cells. Expression of Granzyme B identified activated NK cells. Expression of synaptophysin identified tumor cells. **(B)** Quantification of NK cells and Granzyme B^+^ NK cells per mm^2^ in ONB tumor and stroma (*n* = 38 tumor cores in triplicate). **(C)** NK cell density when comparing patients who presented with and without dural infiltration, top, and Dulgerov stages, bottom. **(D)** Granzyme B^+^ NK cell densities when compared by whether the patient had a post-treatment recurrence, top, and whether their cause of death was ONB specific. All lines are graphed to indicate the median value. For paired comparisons, Wilcoxon matched-pairs signed rank test and for unpaired comparisons, Mann-Whitney U tests were used to test for statistical significance. *p ≤ 0.05, non-significant

Natural killer (NK) cells are involved in innate defense through surveillance of aberrant expression of MHC-I molecules. NK cells can efficiently attack cells that show signals of oncogenic transformation and also represent a promising immunotherapy approach [31, 32]. NK cells were identified in the ONB TIME through the co-localization of CD16 and CD56 in a panel that also contained CD3, Granzyme B, and synaptophysin (Fig. 3A). CD56 is a classic NK cell marker also known as neural cell adhesion molecule and, consistent with prior literature, a large portion of the olfactory neuroblastoma tumor cells also stained CD56 positive [33]. Therefore, a size threshold was used to exclude larger ONB tumor cells from the pool of smaller NK cells. Granzyme B was used as a marker of NK cell activation. NK cells were relatively sparse in the ONB TIME found at a median of 0.81 cells per mm^2^ and Granzyme B^+^ cells were found at a median of 0 cells per mm^2^ and only made up 4% of identified NK cells. There was no difference in NK cell density distribution when comparing the tumor and stroma compartments (Fig. 3B).

NK immune cell distributions were compared with clinical factors of interest. Patients who presented without dural infiltration had more NK cells than those who presented with dural infiltration (medians of 1.64 cells per mm^2^ versus 0.52 cells per mm^2^). Similarly, when compared globally low Dulgerov stage (T1 or T2) tumors presented with more NK cells (medians of 1.47 cells per mm^2^ versus 0.51 cells per mm^2^, *P* = 0.047) (Fig. 3C). This could indicate that NK cells may hinder disease progression and dural infiltration but requires further investigation. However, when looking at activated, Granzyme B^+^, NK cells, these were found in higher abundance in samples where patients went on to have post-treatment recurrences and in patients who passed due to their olfactory neuroblastoma *P* = 0.0221 and *P* = 0.048, respectively) (Fig. 3D). Given the paucity of activated NK cells, this relationship is unclear and requires further investigation. NK cell density distributions did not correlate with any other measured clinical factors including Hyams grade, Kadish stages, primary versus recurrent samples, orbital involvement, biological sex, or age ≥ 65 (Supplementary Tables 7–9).

### MHC expression in ONB tumor cells

**Fig. 4:**
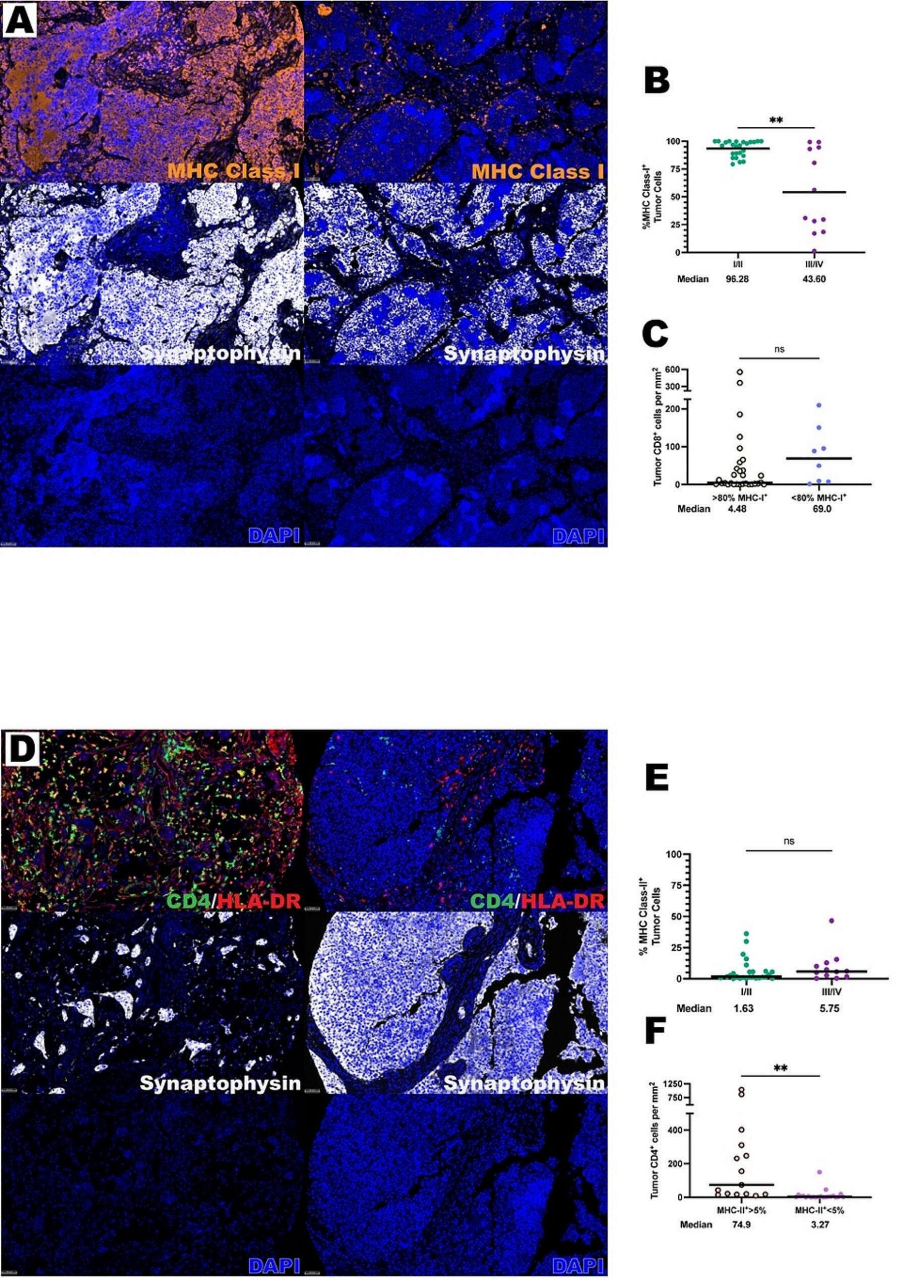
Differential expression of MHC-I and MHC-II exist in the ONB TIME. **(A)** Representative photomicrographs at 20x magnification from two ONBs of merged and single-color immunofluorescence images assessing the presence of MHC-I^+^ cells with a validated panel of six biomarkers, CD8 (yellow, Opal 570), HLA-DR (red, Opal 690), Ki-67 (turquoise, Opal 480), MHC-II (orange, Opal 620), CD4 (green, Opal 520), and synaptophysin (white, Opal 780). Multispectral immunofluorescence images are counterstained with DAPI. Expression of synaptophysin identified tumor cells and MHC-I identified MHC-I^+^ cells. Right column is representative of a high Hyams Grade tumor (Hyams grade III) and the left column is representative of a low Hyams Grade Tumor (Hyams Grade I). **(B)** Quantification of percentage of MHC-I^+^ tumor cells by Hyams Grade and **(C)** MHC-I expression by density of CD8^+^ T cells. **(D)** Representative photomicrographs at 20x magnification from two ONBs of merged and single-color immunofluorescence images assessing the presence of MHC-II^+^ cells and CD4^+^ T cells with a validated panel of six biomarkers, CD8 (yellow, Opal 570), HLA-DR (red, Opal 690), Ki-67 (turquoise, Opal 480), MHC-II (orange, Opal 620), CD4 (green, Opal 520), and synaptophysin (white, Opal 780). Multispectral immunofluorescence images are counterstained with DAPI. Expression of synaptophysin identified tumor cells, HLA-DR identified MHC-II^+^ cells, and CD4 identified CD4^+^ T cells. **(E)** Left column is representative of a high CD4^+^ infiltrated tumor and the right column is representative of a low CD4^+^ infiltrated tumor. **(F)** Quantification of percentage of MHC-II^+^ tumor cells by Hyams Grade and MHC-II expression by density of CD4^+^ T cells. All lines are graphed to indicate the median value. Mann-Whitney U tests were used to test for statistical significance. **p ≤ 0.01, ns, non-significant

Next, we evaluated mechanisms that may be responsible for a stromal predilection of immune cell infiltrate in the ONB TIME. MHC presentation of tumor antigens is critical for T cell mediated immune responses. Indeed, HLA class I presents antigen to T cell receptors (TCR) on CD8^+^ T cells while HLA class II presents antigen to TCRs on CD4^+^ T cells. Therefore, if HLA is absent, T cells cannot engage their TCR and proliferate, which could lead to a relative lack of T cell presence within the tumor parenchyma. Additionally, downregulation of MHC-I has been described in a plethora of cancers often correlated with a worse prognosis [34–36]. Through staining of CD4, CD8, HLA-DR, Ki-67, MHC-I, and synaptophysin, the level of MHC-I^+^ and MHC-II^+^ tumor cells in ONB were interrogated as a potential mechanism responsible for the lack of T cell tumor parenchyma trafficking (Fig. 4A). Interestingly, the majority of ONB samples demonstrated high MHC-I expression, but strikingly decreased MHC-I expression was identified in high Hyams grade samples as compared to low Hyams grade samples (median of 43.60% MHC-I^+^ tumor cells in high Hyams grade versus 96.28% MHC-I^+^ tumor cells in low Hyams grade, *P* < 0.0001) (Fig. 4B). This may indicate that the loss of MHC-I tumor cell expression may represent a mechanism of immune evasion in higher grade ONB. A correlation was not observed between MHC-I expression and the degree of CD8^+^ T cell (Fig. 4C) or CD4^+^ T cell infiltration.

MHC-II molecules are notably found on antigen-presenting cells but may also be expressed by tumor cells. Human leukocyte antigen – DR (HLA-DR) is an isotype of MHC-II that is a ligand for the T cell receptor and upregulated by immune stimulation [37]. Tumor specific MHC-II expression is important for immune surveillance and tumor cell recognition via CD4^+^ TCR engagement of class II-restricted antigens and plays a role in immunotherapy response [38]. Interestingly, in contrast to MHC-I there was low generalized expression of MHC-II observed in ONB tumor cells (Fig. 4D). No difference was observed in MHC-II expression when comparing low and high Hyams grade ONB (Fig. 4E). Interestingly, a relationship between HLA-DR^+^ tumor cell expression and the degree of CD4^+^ T helper cell tumor infiltration was observed. In those ONB tumors with higher HLA-DR^+^ expression, more T helper cell tumor parenchymal infiltration occurred compared to lower MHC-II expressing samples (medians of 74.90 cells per mm^2^ vs. 3.27 cells per mm^2^, *P* < 0.006) (Fig. 4F). Additionally, more CD8^+^ cells were noted in high HLA-DR expressing samples (median 62.29 cells per mm^2^ for high HLA-DR^+^ samples vs. median 2.05 cells per mm^2^ for low HLA-DR^+^ samples, p-value < 0.0001) This indicates that increasing HLA-DR expression in ONB tumor cells may help to drive T cell trafficking into the ONB tumor parenchyma.

### ONB tumor cell chemokine expression

**Fig. 5:**
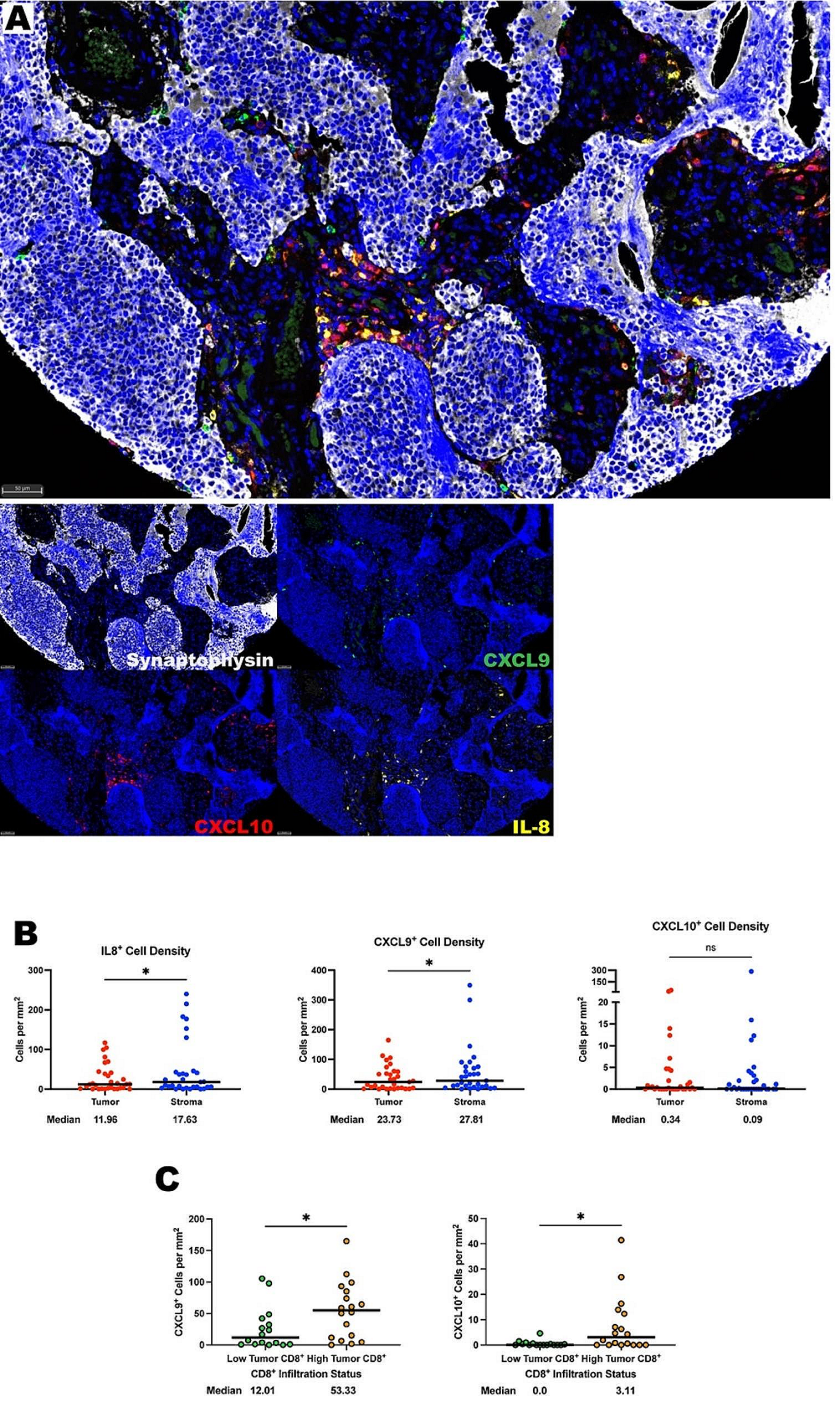
IL-8, CXCL-9, and CXCL10 are found in abundance within the olfactory neuroblastoma TIME. **(A)** Representative photomicrographs at 20x magnification from a low Hyams grade ONB of merged and single-color immunofluorescence images assessing the presence of chemokines with a panel of IL-8 (yellow, Opal 570), CXCL10 (red, Opal 690), CXCL9 (green, Opal 520), and synaptophysin (white, Opal 780). Multispectral immunofluorescence images are counterstained with DAPI. Expression of synaptophysin identified tumor cells, IL-8 identified IL-8^+^ cells, CXCL9 identified CXCL9^+^ cells, and CXCL10 identified CXCL10^+^ cells. **(B)** Quantification of IL-8^+^ cells, CXCL9^+^ cells, and CXCL10^+^ cells per mm^2^ in ONB tumor and stroma (*n* = 32 tumor cores in triplicate). **(C)** Quantification of CXCL9^+^ and CXCL10^+^ cells in the tumor compartment by CD8^+^ tumor infiltration status. All lines are graphed to indicate the median value. For paired comparisons, Wilcoxon matched-pairs signed rank test and for unpaired comparisons, Mann-Whitney U tests were used to test for statistical significance. *p ≤ 0.05, ns, non-significant

Next, we hypothesized that decreased chemokine expression by ONB tumor cells may also contribute to the stromal predilection of immune cells in ONB. Key chemokines including CXCL9 and CXCL10 have been shown to attract CD8^+^ cytotoxic T cells to the tumor microenvironment [17]. We implemented in situ hybridization to detect messenger ribonucleic acid (mRNA) transcripts of CXCL9, CXCL10, and IL-8 along with a synaptophysin immunohistochemical co-stain to identify tumor cells (Fig. 5A). First, CXCL9^+^ cells were found in higher concentrations in the stroma (*P* = 0.0179; Fig. 5B) correlating with the stromal propensity of CD8^+^ cytotoxic T cells from our prior CD8^+^ T cell analysis. CXCL10^+^ cells were fewer in the ONB TIME at medians of 0.34 cells per mm^2^ in the tumor and 0.09 cells per mm^2^ in the stroma with no significant difference between compartments. In our cohort, samples containing more CD8^+^ T cells, ≥ 10 cells per mm^2^, in the tumor parenchyma contained more CXCL9^+^ and CXCL10^+^ tumor cells, indicating that tumor cell expression of these key chemokines may also drive tumor parenchymal infiltration of CD8^+^ T cells (Fig. 5C).

Interleukin-8 has been shown to recruit a suppressive set of myeloid cells [20]. More IL-8^+^ cells were found in the stroma when compared to the tumor (median of 17.63 cells per mm^2^ vs. median of 11.96 cells per mm^2^, *P* = 0.0168) which correlated with prior findings of more MDSCs in the stromal compartment (Fig. 5B). However, the degree of tumor parenchymal IL-8 expressing cells did not correlate with the degree of PMN-MDSC tumor parenchymal infiltration (Supplementary Fig. 6).

## Discussion

In this study, we hypothesized that an in-depth evaluation of the ONB TIME would define mechanisms of immune evasion in high-grade ONB and identify rational immunotherapeutic strategies. Given the rare nature of this tumor, our cohort is very large and captures tumors from all stages and grades. This study identified a significant T cell and myeloid cell infiltrate; however, these cells were largely identified in the stroma rather than in the tumor parenchyma. Immune cell intraparenchymal infiltration is important for tumor cell surveillance and may be driven by tumor cell MHC and chemokine expression. Interestingly, MHC-I was highly expressed in low-grade ONB but poorly expressed in high-grade ONB which may be an important mechanism of immune evasion in high-grade ONB. In contrast, MHC-II (HLA-DR) was poorly expressed in ONB, and critically, tumors with increased MHC-II expression demonstrated significantly more T cell intraparenchymal infiltration (Fig. 4). This indicates that a lack of HLA-DR expression in ONB in part drives the stromal predilection of T cells and that strategies to increase MHC-II tumor cell expression may improve intraparenchymal localization. Additionally, another key finding of this study was that ONB with high intra-tumoral CD8^+^ T cell infiltrates demonstrated significantly higher CXCL9 and CXCL10 tumor cell expression. These data collectively suggest that strategies that increase ONB tumor cell expression of MHC-II, CXCL9, and CXCL10 may be particularly beneficial for T cell recruitment and activation in ONB immunotherapeutic approaches [39, 40]. Like CXCL9, CXCL10 and CXCL11 expression, HLA-DR expression on tumor cells is tightly linked to IFN-γ signaling. Given the very high constitutive expression of HLA class I observed in these tumors, HLA class II expression may act as a surrogate measure for the magnitude of T cell-derived IFN-γ in the local microenvironment. Given the close mechanistic association between IFN-γ signaling, more study is needed to understand the true mechanisms driving the heterogeneous HLA class II expression and T cell infiltration observed in these tumors.

This study also identified T_regs_ as a potential mechanism of tumor immune suppression in ONB. This is supported by spatial analysis which demonstrated that T_regs_ are significantly closer in proximity to non-proliferating CD8^+^ than proliferating CD8^+^ cytotoxic T cells, suggesting a potential suppressive effect on the CD8^+^ T cell proliferation in ONB. One of key mediators driving T cell polarization into T_regs_ is TGF-β. Interestingly, increased TGF-β signaling has been previously linked to shorter disease-free survival in ONB [28]. Indeed, one currently available ONB-specific interventional trial is available in the United States (NCT05012098); investigating a bifunctional fusion protein targeting TGF-β and PD-L1 in recurrent and metastatic ONB. No results are currently available from this clinical trial. PD-L1 expression has been previously demonstrated by our group and others in approximately 40% of ONB [22, 23]. This present study also identified significant expression of PD-1 in CD8^+^ T cells in particular, and to a lower degree in CD4^+^ and T_regs_. Collectively these findings further support targeting the PD-1/PD-L1 and TGF-β signaling pathways for ONB.

Evaluation of the ONB myeloid compartment identified a significant TIME infiltration of myeloid cells including tumor associated macrophages, PMN, and monocytes. Similar to T cells, there was a significant stromal predilection for myeloid cells with poor intraparenchymal infiltration. A subset of myeloid cells termed MDSCs has been shown to dampen immune tumor recognition in multiple tumor types [41, 42]. Interestingly, no PMN-MDSCs were identified within the tumor parenchyma of any Kadish A/B patients. This is in contrast to Kadish C/D patients in which a significant increase in intra-tumoral infiltration of PMN-MDSCs was observed (Fig. 2C). This suggests that intraparenchymal recruitment of this PMN-MDSC cell type may be critical for ONB disease progression and immune evasion.

While T cell and myeloid cells were consistently observed in ONB, NK cells were infrequently identified in the ONB TIME. As previously mentioned, NK cells play an important role in innate defense through destruction of tumor cells with aberrant MHC-I expression. While the majority of ONB tumors highly express MHC-I, a significant reduction was observed in MHC-I expression in high Hyams grade disease. Thus, patients with high Hyams grade disease may be particularly susceptible to NK cell-based approaches. Given the low levels of endogenous NK cells in the ONB TIME these would need to be recruited from the circulation or through adoptive transfer approaches [43, 44]. For example, use of an interleukin-15 superagonist could aid in activation of immune cell subsets and increase release of inflammatory cytokines [45]. Another possibility would be to combine immunotherapy approaches with radiation or chemotherapy which has been shown to increase MHC class I presentation in other tumor types [46–48].

In this study, use of a large collection of clinically annotated samples of this rare tumor identified newly described correlations between low MHC-I expression, increased PMN-MDSC intraparenchymal infiltration, and an increased number of parenchyma localized Ki-67^+^ T helper cells in high grade/stage ONB. Extensive evaluation with additional clinical factors did not identify correlations between the T cell, myeloid cell, NK cell, or MHC expression data and many additional important clinical factors (Supplementary Tables 1–9). Limitations of this study include its retrospective nature and the procurement of patient samples from a single institution. Future prospective and multi-institutional studies will be helpful in validating and advancing the results of this study. While this study did include recurrent samples, it did not include samples from metastatic ONB, and this is an ONB subset that warrants further study. Multi-spectral immunofluorescence is also limited in the number of markers that can be simultaneously evaluated using a single panel, thus further studies are needed to evaluate and delineate the increasing complexity of immune cell sub-populations. Advancements and emerging spatial transcriptomics approaches with formalin-fixed paraffin embedded tissue will allow for a more in-depth characterization at the single cell level in the future. Ongoing multi-institutional and international collaborations in the field of rare tumors, like ONB, will translate these findings into clinical practice.

## Conclusion

This study of the ONB TIME demonstrated significant T cell infiltration with a stromal predilection, presence of myeloid-derived suppressor cells, and sparse natural killer cells. A striking decrease was observed in MHC-I expression in high-grade olfactory neuroblastoma compared to low-grade disease, representing a mechanism of immune evasion in high-grade disease. The stromal predilection appears to be driven by low tumor cell MHC class II (HLA-DR), CXCL9, and CXCL10 expression as increased tumor cell expression of these correlated with significant increases in parenchymal T cell infiltration. Immunotherapeutic strategies that augment tumor cell expression of MHC class II, CXCL9, and CXCL10 may improve parenchymal trafficking of immune effector cells in ONB and augment immunotherapeutic responses.

## Methods

### Case Selection

A tissue microarray containing forty-seven clinically annotated ONB samples in triplicate was obtained along with institutional review board approval from Johns Hopkins. A retrospective chart review of this patient cohort was conducted with clinical variables of interest collected including patient demographics; Hyams grade; Kadish stage; Dulguerov stage; last documented clinical status; presenting symptoms; presence of dural invasion, intracranial extension, or orbital involvement; treatment characteristics; existence of post-treatment recurrence and cause of death when applicable.

Multiplex Panel Validation.

The dilution of each antibody specific to formalin fixed paraffin embedded (FFPE) ONB type was determined using monoplex immunohistochemistry (IHC) and then subsequent monoplex immunofluorescence (IF). Using chromogen 3’-3’ diaminobenzidine tetrahydrochloride hydrate detection (BOND Polymer Refine Detection, Leica Biosystems, New Castle Upon Tyne, UK; #DS9800) optimal marker strength and specificity was determined and this dilution was used as a starting point for IF. IHC positive control tissue for antibody validation, either normal tonsil or normal lung tissue, was chosen based on previously identified protein of interest expression. Unstained ONB tissue (4–5 μm sections) served as a negative control for all validation steps. Once optimal IF dilutions were established, multiple combinations of antibodies were tested to determine the most appropriate staining order for the final creation of each multispectral immunofluorescence (mxIF) panel (Supplementary Tables 10–13).

The T cell panel consisted of antibodies against CD4, CD8, FOXP3, PD-1, Ki-67 and Synaptophysin. Staining was completed in this order. This T cell panel revealed the presence of T-regulatory cells, CD4^+^ T-helper cells, and CD8^+^ cytotoxic T cells. T-regulatory cells were simultaneously positive for CD4 and FOXP3 markers. CD4^+^ T-helper cell phenotypes were CD4 + and CD8^−^/FOXP3^−^, while CD8^+^ cytotoxic T cell phenotypes were CD8^+^ and CD4^−^/FOXP3^−^. Positive Ki-67 staining denoted proliferating cells. PD-1 stain allowed for identification of PD-1^+^ T cells.

The myeloid derived suppressor cell (MDSC) panel was comprised of antibodies against CD15, CD68, CD11b, CD14, HLA-DR and Synaptophysin. Staining was completed in this order. The myeloid panel allowed for the recognition of macrophages, monocytes, and polymorphonuclear leukocytes (PMNs). High expressing CD68 cells were considered tumor associated macrophages. Monocyte-MDSC phenotypes were CD11b^+^/CD14^+^/CD15^−^/HLA-DR^low/−^ while PMN-MDSC phenotypes were CD11b^+^/C14^−^/CD15^+^/HLA-DR^low/−^.

The NK cell panel was comprised of antibodies against CD3, CD56 (NCAM1), CD16, Granzyme B, and Synaptophysin. Staining was completed in this order. A phenotype of CD16^+^/CD56^+^/CD3^−^ was used to identify NK cells. As ONB cells also express CD56, a decreased size threshold relative to tumor cells was used to distinguish NK cells from tumor cells. Granzyme B positivity denoted activated NK cells. The MHC panel contained antibodies against CD4, CD8, HLA-DR, MHC Class I, Ki-67, and Synaptophysin. Staining was completed in that order. HLA-DR was used as a surrogate marker for MHC Class II as it is one isotype of the MHC Class II complex.

All slides for each of these four panels were counterstained with 4′,6-diamidino-2-phenylindole (DAPI) to identify cell nuclei. Synaptophysin was used as an ONB tumor cell marker and it delineated tumor parenchyma from stroma.

### Tissue preparation, multiplex staining, and high-resolution scanning

Slides were first baked at 60ºC for 30 min and soaked in Bond Dewax Solution (Leica Biosystems, #AR9222) at 72ºC followed by rehydration with 100% ethanol. Leica BOND Rx autostainer (Leica Biosystems Melbourne Pty Ltd, Melbourne, Australia) was used for deparaffinization and staining of all tissue.

Standardized staining protocols (Perkin Elmer platform), using the Leica Bond Rx automated system described above, were completed with 4 μm FFPE sections of ONB tissue. Heat induced epitope retrieval was executed for all antibodies except CD15 by heating slides to 95ºC and treating with paired BOND Epitope Retrieval (ER) solutions, either citrate-based ER1 (Leica Biosystems, #AR9961) or EDTA-based ER2 (Leica Biosystems, #AR9640). Primary antibody was applied for 60 min, followed by secondary HRP-conjugated antibody for 30 min, and finalized by fluorescent signal amplification for every protein target. OPAL (Akoya Biosciences) multiplex kit consisting of OPAL-480, 520, 570, 620, 690, 780 conjugates allowed for the evaluation of up to six colors simultaneously. Except for Opal 780 made at 1:50 dilution, all opals were made at a 1:150 dilution. Opal 780 was combined with TSA-DIG at a 1:100 dilution. After staining, slides were cover slipped with the Leica CV5030 automated glass cover slipper (Leica Biosystems, Nussloch, Germany Ltd) and scanned at 40x or 20x magnification with suitable exposure times using PerkinElmer Vectra Polaris for the creation of high-resolution digital images. Several samples had to be excluded in cases when there was too little tissue on the TMA for meaningful analysis.

### RNAScope

The expression of IL-8, CXCL-9, and CXCL-10 genes in human ONB tumors were detected by staining 5 μm FFPE tissue array sections with the RNAscope 2.5 LS probes Hs-CXCL9 (ACD, Cat# 440,168), Hs-IL8-C2 (ACD, Cat# 310,388-C2), and HS-CXCL10-C3 (ACD, Cat# 311,858-C3),with the RNAscope® LS Multiplex Fluorescent Assay (ACD, Cat# 322,800) using the Bond RX auto-stainer (Leica Biosystems) with a tissue pretreatment of 30 min at 100 °C with Bond Epitope Retrieval Solution 2 (Leica Biosystems) and a 1:750 dilution of TSA-OPAL520, TSA-OPAL570 and TSA-OPAL690 (AKOYA Biosciences®), respectively. To delineate the tumor parenchyma from the stroma and isolate the specific compartment where the chemokines are expressed, sections where subsequently IHC stained with rabbit anti-human Synaptophysin (YE269) antibody (Abcam Ref# ab32127) at a 1:2,500 dilution for 30 min using the Bond Polymer Refine Kit (Leica Biosystems, Cat# DS9800) minus Post Primary reagent, DAB, and Hematoxylin with a 1:100 dilution of OPAL TSA-DIG reagent for 10 min. and a 1:25 dilution of the Opal Polaris 780 antibody for 60 min. The RNAscope® 3-plex LS Multiplex Negative Control Probe (Bacillus subtilis dihydrodipicolinate reductase (dapB) gene in channels C1, C2, and C3, Cat# 320,878) followed by IHC with no primary antibody was used as an ISH and IHC negative control. Slides were digitally imaged using an PhenoImager™ HT Scanner (AKOYA Biosciences) (Supplementary Table 14).

### Image analysis

Images were analyzed using the HALO® (Indica Labs, Albuquerque, NM, USA) platform v3.5, which allows for cell phenotype quantification, identification of marker co-localization, along with spatial and infiltration analysis. First, slides were annotated into tumor parenchyma and stroma using the HALO® random forest classifier based on positive synaptophysin staining and tumor and stroma compartments confirmed with a hematoxylin and eosin (H&E) stained tissue microarray when necessary. Histologic areas of bone, bone marrow, and blood vessels, identified using H&E stains, along with auto fluorescent tissue, were manually excluded in the final analysis. Cell size, nuclear to cytoplasmic ratio, nuclear segmentation, and individual biomarker fluorescence intensity thresholds were manually set to identify cellular phenotypes of interest. These were calibrated for each independent core on the TMA to account for variability in staining uptake. Samples with insufficient tissue in each panel were excluded from analysis. Quantification analyses were run using the HALO® Highplex FL analysis algorithm v4.1.3. Nearest neighbor analyses, to determine the average distance and number of unique neighbors between two cell populations, and proximity analyses, for calculation of the number of cells within a specified distance of a given cell of interest, were conducted. HALO® density heat maps of all immune cell compartments were created to visually compare immune infiltrate patterns.

### Statistical analysis

Wilcoxon signed rank tests were used to detect statistical differences between immune cell densities in the tumor parenchyma and stroma. Mann-Whitney tests were used to determine statistical significance between clinical factors of interest. A P value significance threshold of < 0.05 was employed in all cases. All statistics and were conducted and graphs prepared using GraphPad Prism version 9.3.1.

## Electronic supplementary material

Below is the link to the electronic supplementary material.

Supplementary Material 1

Supplementary Material 2

Supplementary Material 3

Supplementary Material 4

## Data Availability

Data are available upon request.

## Abbreviations

CXCL9: C-X-C motif ligand 9
CXCL10: C-X-C motif ligand 10
IL-8: Interluekin-8
MHC-I: Major histocompatibility complex class I
MHC-II: Major histocompatibility complex class II
mxIF: Multispectral immunofluorescence
MDSCs: Myeloid-derived suppressor cells
NK: Natural killer cells
ONB: Olfactory neuroblastoma
PD-L1: Programmed cell death-ligand 1
T_regs_: Regulatory T cells
TAMs: Tumor associated macrophages
TIME: Tumor immune microenvironment
TGF-β: Transforming growth factor-beta
