# Correction to: Augmentation of tumor expression of HLADR, CXCL9, and CXCL10 may improve olfactory neuroblastoma immunotherapeutic responses

**DOI:** 10.1186/s12967-024-05514-y

**Published:** 2024-09-11

**Authors:** Riley M. Larkin, Diana C. Lopez, Yvette L. Robbins, Wiem Lassoued, Kenneth Canubas, Andrew Warner, Baktiar Karim, Ksenia Vulikh, James W. Hodge, Charalampos S. Floudas, James L. Gulley, Gary L. Gallia, Clint T. Allen, Nyall R. London

**Affiliations:** 1 Sinonasal and Skull Base Tumor Program, Surgical Oncology Program, Center for Cancer Research, National Cancer Institute, National Institutes of Health, Bethesda, MD USA; 2 University of Miami Miller School of Medicine, Miami, MD USA; 3 Department of Otolaryngology-Head and Neck Surgery, Johns Hopkins University School of Medicine, Baltimore, MD USA; 4 Section on Translational Tumor Immunology, Center for Cancer Research, National Cancer Institute, National Institutes of Health, Bethesda, MD USA; 5 Center for Immuno-Oncology, Center for Cancer Research, National Cancer Institute, National Institutes of Health, Bethesda, MD USA; 6 Molecular Histopathology Laboratory, Frederick National Laboratory for Cancer Research, Frederick, MD USA; 7 Department of Neurosurgery, Johns Hopkins University School of Medicine, Baltimore, MD USA

**Correction to: Journal of Translational Medicine (2022)22:524**

10.1186/s12967-024-05339-9

Following publication of the original article [1], we have been notified that there was a mistake in Copyright line.

It is now as follows: © The Author(s) 2024. Open Access. It should be as follows: This is a U.S. Government work and not under copyright protection in the US; foreign copyright protection may apply 2024.

The original article was updated.
