# Supplementary material for: Augmentation of tumor expression of HLA-DR, CXCL9, and CXCL10 may improve olfactory neuroblastoma immunotherapeutic responses
Source: J Transl Med. 2024 May 31;22:524. doi: 10.1186/s12967-024-05339-9 (PMC11140921; doi:10.1186/s12967-024-05339-9)
Supplement: Supplementary file 1 — Supplementary Material 1 [file 12967_2024_5339_MOESM1_ESM.docx]

**Figure 1. T cell Panel and Quantification Analysis**

**
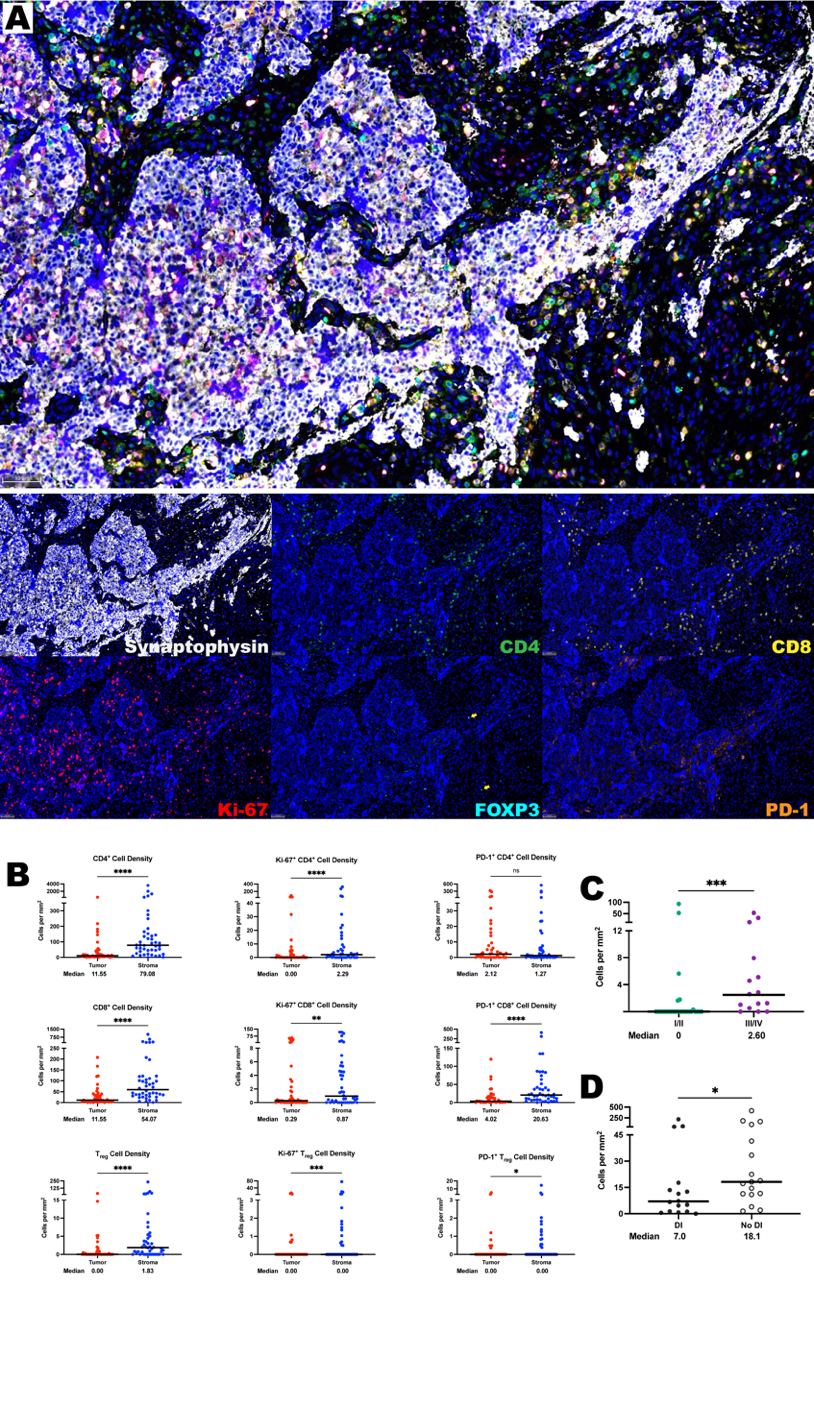
**

**Figure 1. T cells are found in abundance within the olfactory neuroblastoma tumor immune microenvironment, most often within the stroma.** **(A)** Representative photomicrographs at 20x magnification from a high Hyams grade ONB of merged and single-color immunofluorescence images assessing the presence of T cells with a validated panel of six biomarkers, CD8 (yellow, Opal 570), CD4 (green, Opal 520), FOXP3 (turquoise, Opal 480) indicated by yellow arrows, Ki67 (red, Opal 690), PD-1 (orange, Opal 620), and synaptophysin (white, Opal 780). Multispectral immunofluorescence images are counterstained with DAPI. Expression of CD4 without CD8 and FOXP3 identified CD4^+^ T helper cells, while expression of CD8 without CD4 and FOXP3 identified CD8^+^ cytotoxic effector T cells. Co-localization of CD4 and FOXP3 without CD8 identified T_regs_. T cells with positive Ki-67 nuclear expression were proliferating, and PD-1 positivity denoted PD-1^+^ T cells. Expression of synaptophysin identified tumor cells. **(B)** Quantification of CD4^+^ T cell, CD8^+^ T cell, and T_reg_ cell density (cells per mm^2^), as well as proliferating and PD-1^+^ subcategories of these are compared between ONB tumor and stroma (n = 44 tumor cores). **(C)** Quantification of tumor specific Ki-67^+^ CD4^+^ T helper cells compared by Hyams Grade. **(D)** Quantification of tumor specific CD4^+^ T helper cells when comparing patients who presented with and without dural infiltration. For paired comparisons, Wilcoxon matched-pairs signed rank test and for unpaired comparisons, Mann-Whitney U tests were used to test for statistical significance. All lines are graphed to indicate the median value. **p*≤0.05, **p≤0.01, ****p*≤0.001, *****p*≤0.0001, ns, non-significant.

**Figure 2. MDSC Panel and Quantification Analysis**


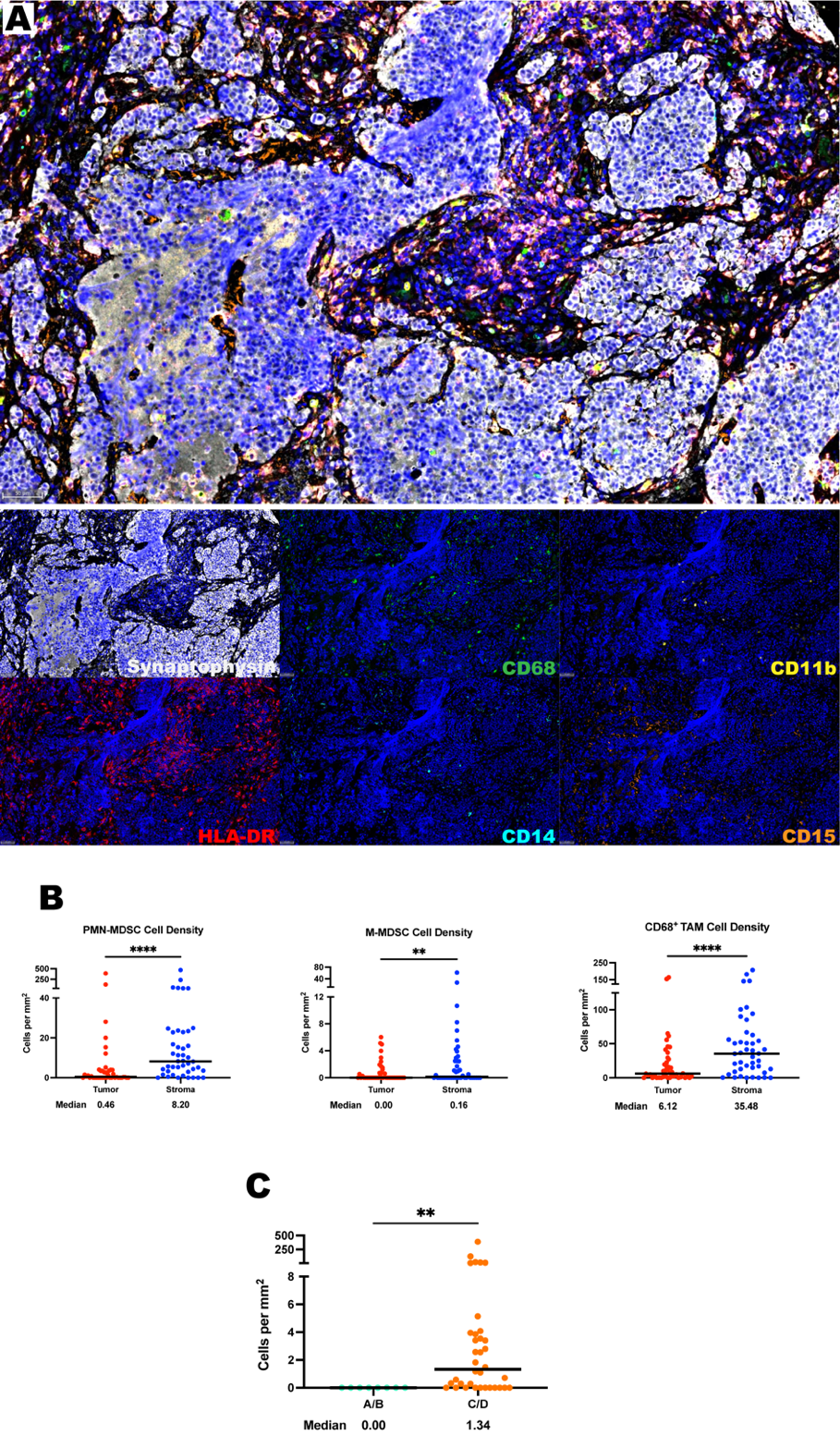


**Figure 2.** **Myeloid cells are found in abundance within the ONB TIME, most often within the stroma.** **(A)** Representative photomicrographs at 20x magnification from a low Hyams Grade ONB of merged and single-color immunofluorescence images assessing the presence of myeloid cells with a validated panel of six biomarkers, CD11b (yellow, Opal 570), HLA-DR (red, Opal 690), CD14 (turquoise, Opal 480), CD15 (orange, Opal 620), CD68 (green, Opal 520), and synaptophysin (white, Opal 780). Multispectral immunofluorescence images are counterstained with DAPI. High expression of CD68 identified tumor associated macrophages. Co-localization of CD11b and CD14 without CD15 and little to no HLA-DR identified M-MDSCs. Co-localization of CD11b and CD15 without CD14 and little to no HLA-DR identified PMN-MDSCs. Expression of synaptophysin identified tumor cells. **(B)** Quantification of PMN-MDSCs, M-MDSCs, and CD68^+^ TAMs per mm^2^ in ONB tumor and stroma (n = 44 tumor cores in triplicate). **(C)** Tumor PMN-MDSC cell density compared by Kadish stage – Low Kadish Stage (A/B) versus High Kadish Stage (C/D). All lines are graphed to indicate the median value. For paired comparisons, Wilcoxon matched-pairs signed rank test and for unpaired comparisons, Mann-Whitney U tests were used to test for statistical significance. **p≤0.01, *****p*≤0.0001

**Figure 3. NK Panel and Quantification Analysis**

**
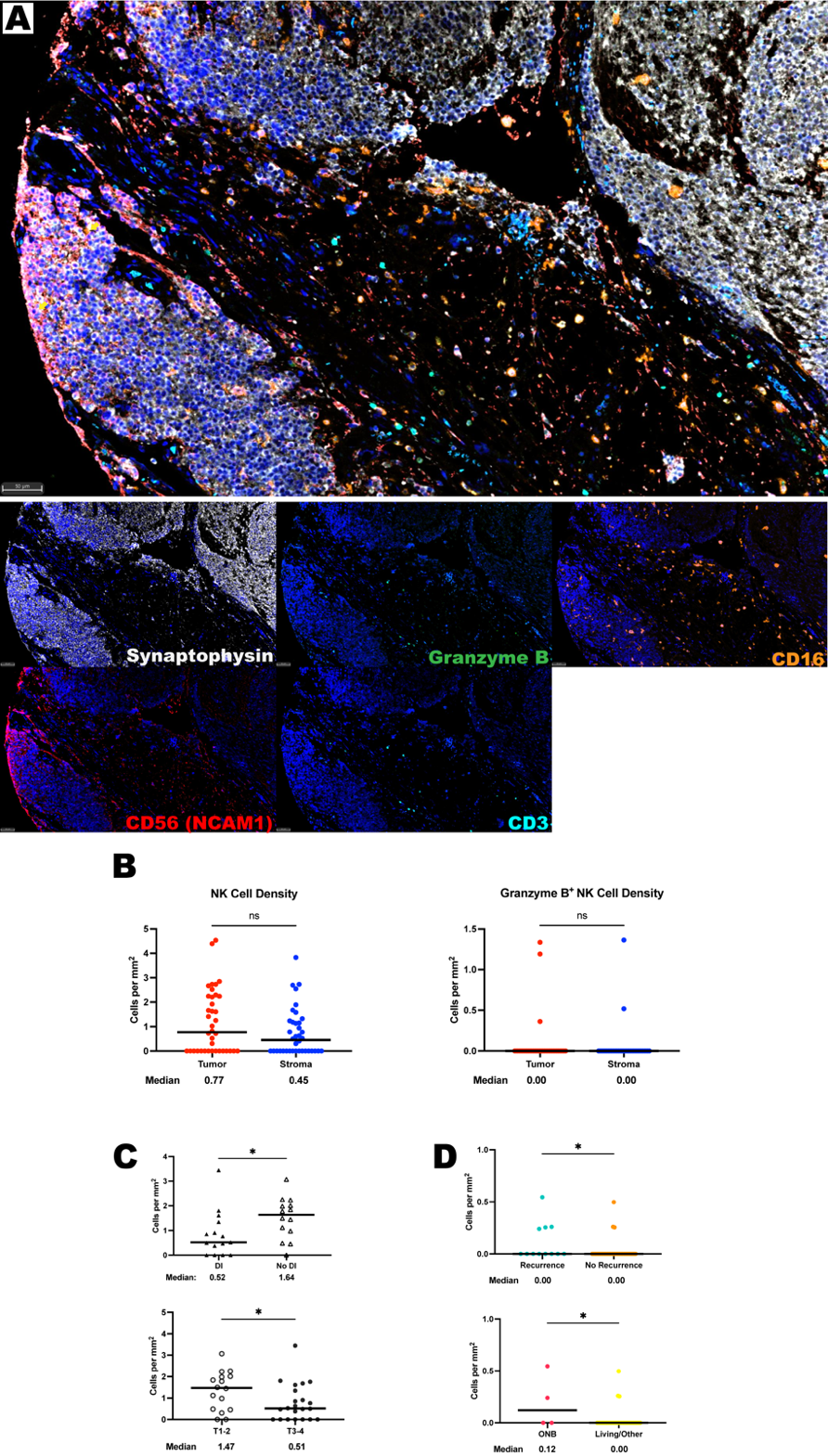
**

**Figure 3**. **NK cells are sparsely found within the ONB TIME.** **(A)** Representative photomicrographs at 20x magnification from a low Hyams Grade ONB of merged and single-color immunofluorescence images assessing the presence of NK cells with a validated panel of five biomarkers, CD16 (orange, Opal 620), CD56 (red, Opal 690), CD3 (turquoise, Opal 480), Granzyme B (green, Opal 520), and synaptophysin (white, Opal 780). Multispectral immunofluorescence images are counterstained with DAPI. Co-localization of CD16 and CD56 without CD3 and a decrease size threshold compared to tumor cells identified NK cells. Expression of Granzyme B identified activated NK cells. Expression of synaptophysin identified tumor cells. **(B)** Quantification of NK cells and Granzyme B^+^ NK cells per mm^2^ in ONB tumor and stroma (n = 38 tumor cores in triplicate). **(C)** NK cell density when comparing patients who presented with and without dural infiltration, top, and Dulgerov stages, bottom. **(D)** Granzyme B^+^ NK cell densities when compared by whether the patient had a post-treatment recurrence, top, and whether their cause of death was ONB specific. All lines are graphed to indicate the median value. For paired comparisons, Wilcoxon matched-pairs signed rank test and for unpaired comparisons, Mann-Whitney U tests were used to test for statistical significance. **p*≤0.05, non-significant.

**Figure 4. Major Histocompatibility Complex Panel and Quantification Analysis**

**
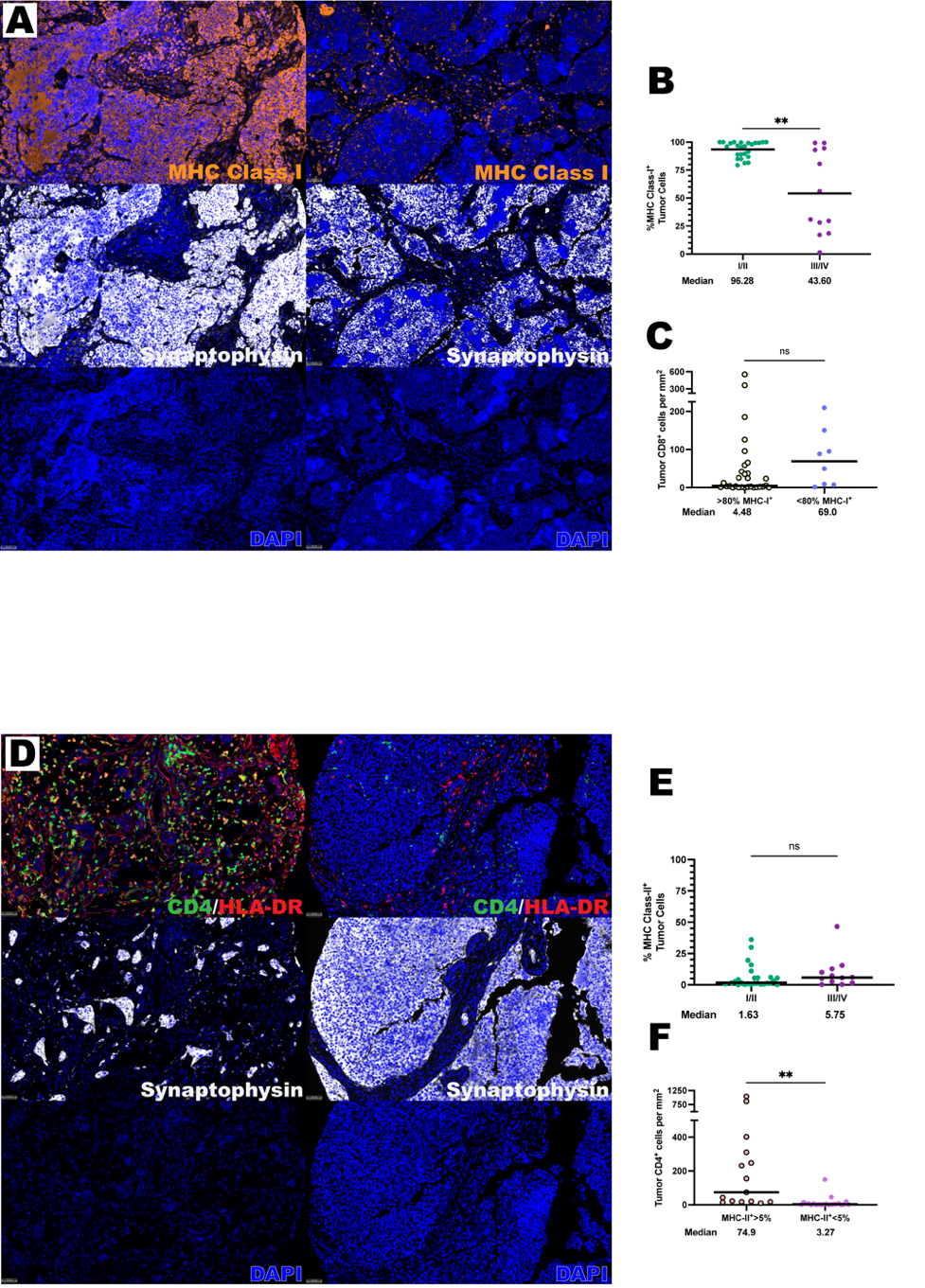
**

**Figure 4. Differential expression of MHC-I and MHC-II exist in the ONB TIME.** **(A)** Representative photomicrographs at 20x magnification from two ONBs of merged and single-color immunofluorescence images assessing the presence of MHC-I^+^ cells with a validated panel of six biomarkers, CD8 (yellow, Opal 570), HLA-DR (red, Opal 690), Ki-67 (turquoise, Opal 480), MHC-II (orange, Opal 620), CD4 (green, Opal 520), and synaptophysin (white, Opal 780). Multispectral immunofluorescence images are counterstained with DAPI. Expression of synaptophysin identified tumor cells and MHC-I identified MHC-I^+^ cells. Right column is representative of a high Hyams Grade tumor (Hyams grade III) and the left column is representative of a low Hyams Grade Tumor (Hyams Grade I). **(B)** Quantification of percentage of MHC-I^+^ tumor cells by Hyams Grade and **(C)** MHC-I expression by density of CD8^+^ T cells. **(D)** Representative photomicrographs at 20x magnification from two ONBs of merged and single-color immunofluorescence images assessing the presence of MHC-II^+^ cells and CD4^+^ T cells with a validated panel of six biomarkers, CD8 (yellow, Opal 570), HLA-DR (red, Opal 690), Ki-67 (turquoise, Opal 480), MHC-II (orange, Opal 620), CD4 (green, Opal 520), and synaptophysin (white, Opal 780). Multispectral immunofluorescence images are counterstained with DAPI. Expression of synaptophysin identified tumor cells, HLA-DR identified MHC-II^+^ cells, and CD4 identified CD4^+^ T cells. **(E)** Left column is representative of a high CD4^+^ infiltrated tumor and the right column is representative of a low CD4^+^ infiltrated tumor. **(F)** Quantification of percentage of MHC-II^+^ tumor cells by Hyams Grade and MHC-II expression by density of CD4^+^ T cells. All lines are graphed to indicate the median value. Mann-Whitney U tests were used to test for statistical significance. **p≤0.01, ns, non-significant.

**Figure 5. RNAScope Panel and Quantification Analysis**

**
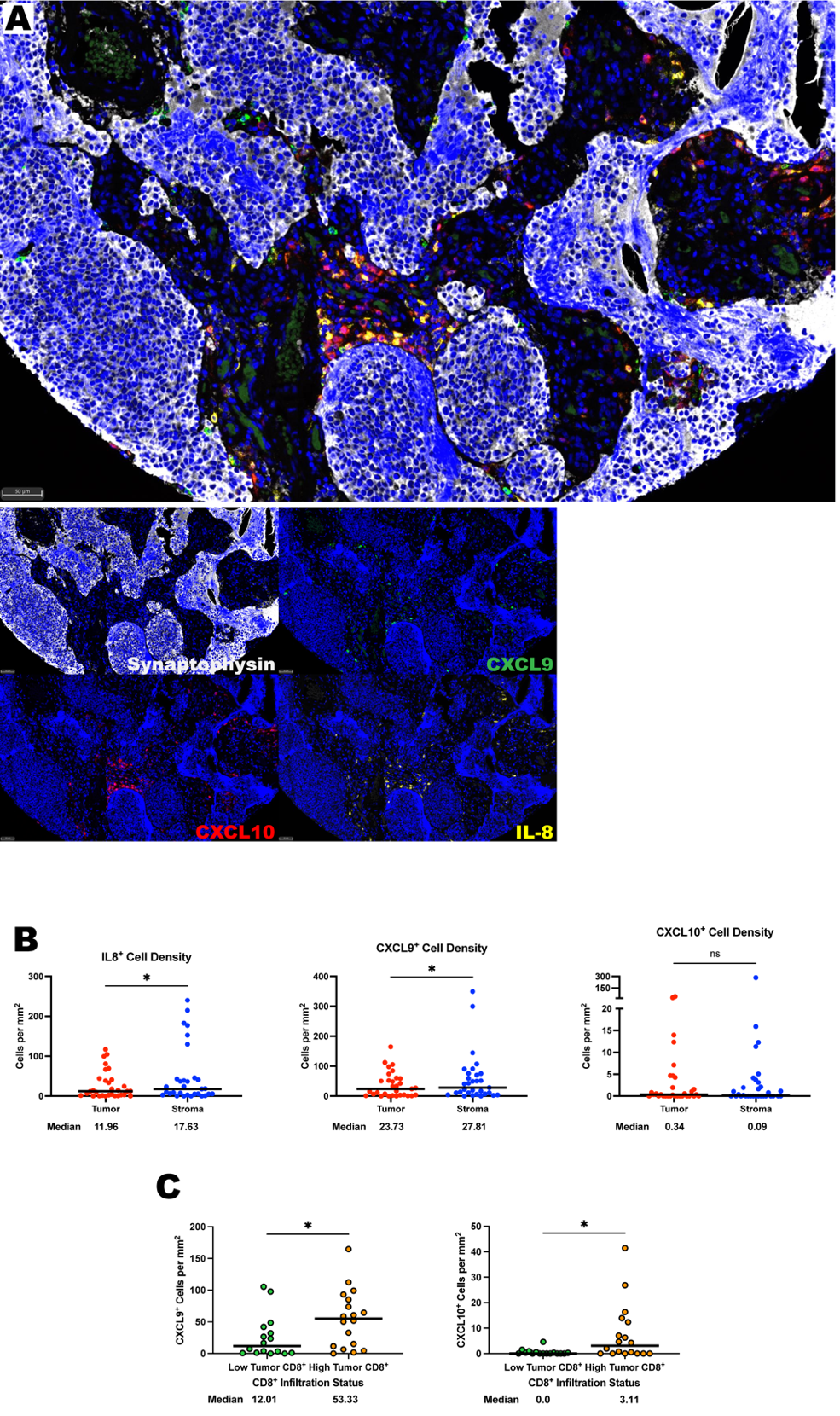
**

**Figure 5.** IL-8, CXCL-9, and CXCL10 are found in abundance within the olfactory neuroblastoma TIME. **(A)** Representative photomicrographs at 20x magnification from a low Hyams grade ONB of merged and single-color immunofluorescence images assessing the presence of chemokines with a panel of IL-8 (yellow, Opal 570), CXCL10 (red, Opal 690), CXCL9 (green, Opal 520), and synaptophysin (white, Opal 780). Multispectral immunofluorescence images are counterstained with DAPI. Expression of synaptophysin identified tumor cells, IL-8 identified IL-8^+^ cells, CXCL9 identified CXCL9^+^ cells, and CXCL10 identified CXCL10^+^ cells. **(B)** Quantification of IL-8^+^ cells, CXCL9^+^ cells, and CXCL10^+^ cells per mm^2^ in ONB tumor and stroma (n = 32 tumor cores in triplicate). **(C)** Quantification of CXCL9^+^ and CXCL10^+^ cells in the tumor compartment by CD8^+^ tumor infiltration status. All lines are graphed to indicate the median value. For paired comparisons, Wilcoxon matched-pairs signed rank test and for unpaired comparisons, Mann-Whitney U tests were used to test for statistical significance. **p*≤0.05, ns, non-significant.
