# Supplementary material for: Augmentation of tumor expression of HLA-DR, CXCL9, and CXCL10 may improve olfactory neuroblastoma immunotherapeutic responses
Source: J Transl Med. 2024 May 31;22:524. doi: 10.1186/s12967-024-05339-9 (PMC11140921; doi:10.1186/s12967-024-05339-9)
Supplement: Supplementary file 3 — Supplementary Material 3 [file 12967_2024_5339_MOESM3_ESM.docx]

We would like to thank the editor and reviewers for their time and efforts in reviewing our manuscript and for the opportunity to submit a revised manuscript. We have incorporated the reviewers’ comments and included a point-by-point response. This manuscript is significantly improved as a result.

**Reviewer #1: Larkin et al., present a nice characterization study of the immune microenvironment across one of the largest collections of human olfactory neuroblastoma (ONB). Utilizing a tissue-microarray setup paired with multispectral immunofluorescence, the authors analyze multiple aspects of the tumor immune microenvironment, including the lymphoid, myeloid, and natural killer cell compartments. They find that lymphoid immune infiltration is largely localized to the tumor stroma, and that there is a significant increase in lymphoid cells in high grade tumors. Similarly, myeloid cells are nearly exclusively in the stroma, and myeloid-derived suppressor cells are enriched in Kadish stages C/D. Natural killer cells are very rare across a majority of the ONBs sampled here. Interestingly, the authors observed a decrease in MHC class I expression in high grade tumors, and furthermore observed that tumors with higher MHC class II expression had higher amounts of CD4 T cells.
Finally, the authors performed RNA-scope across their entire tumor dataset, and found that CXCL9 and CXCL10 both correlate with a higher amount of CD8 T cell infiltration into the tumor, suggesting a potential targetable signaling pathway.

This is a descriptive study, using a retrospective staining and chart review of 47 ONB cases. The report provides insights into the immune microenvironment of ONB, a rare and incompletely understood tumor. With the rise of immunotherapy, including an ONB trial currently in progress, the results presented here are important to elucidate the tumor immune environment within ONB to understand how patients may or may not respond to newer treatments. Overall, the paper is well written, figures are reasonable quality and conclusions are generally supported by data.**

Thank you for the positive comments regarding our study and its translational importance. **Major comments:

1. Throughout the study, cytotoxic T cells are defined as CD8 positivity in the absence of CD4 or FOXP3. While CD8 memory and stem-like CD8 T cells are typically less prevalent in the TIME, the authors should elaborate on how they might exclude the possibility that the CD8 T cells they observe in their staining are not memory or stem-like subsets, and instead have effector functions? Staining for an effector protein/cytokine may help to reliably classify these.**

The authors agree that not all CD8^+^ T cells are effector T cells and some CD8^+^ T cells may represent memory or stem-like subsets. We also agree that memory or stem-like lineages only represent a fraction of CD8^+^ T cells and when the stem-like or memory subsets are further subdivided some groups retain their cytotoxic activity (PMID: 37821656, 37907738). Multi-spectral immunofluorescence is unfortunately limited in the number of markers that can be simultaneously assessed. Our goal was to broadly define the T cell compartment with this panel assessing CD4+, CD8+, and FOXP3+ markers simultaneously. Further delineation of CD8+ subpopulations would require a new multispectral immunofluorescence panel and is an area we would like to pursue in future research studies. We have further addressed this point in the manuscript Discussion lines 375-376 in the marked revised manuscript file.

**2. The authors define tumor as being synaptophysin positive, and stroma as being synaptophysin negative. It is important to note that synaptophysin is a neural marker. While this classification system may work well for many low grade ONBs, it has been shown that many ONBs, especially more aggressive, higher grade tumors, have significantly fewer neuronal components (Classe et al., 2018), i.e. a less-neuronal phenotype. Therefore, it is possible that some portions of their sample, currently defined as stroma, actually represent less differentiated tumor that does not express synaptophysin. If this is the case, there may be some tumors with higher immune infiltration than currently accounted for. Would the authors be able to use some reliable markers for stroma on adjacent tissue sections for each tumor (especially higher grade, less differentiated tumors), to confirm/validate approximate locations of tumor and stroma?**

We thank the reviewer for this comment and agree that it is important to have a standardized method of delineating tumor and stroma aside from synaptophysin expression. We would like to direct the reviewer’s attention to the methods section discussing use of a hematoxylin and eosin-stained tissue microarray. The H&E stain was also used as an adjunct to delineate tumor versus stroma and to confirm the compartments if there was any doubt in the immunofluorescence staining. We have adjusted the language in the manuscript to make this point more clear. Please see lines 482-484.

**3. Could the authors comment on the location from which each of the ONB tissue sections was obtained (if available)? For example, different anatomic locations (intra-nasal vs. intra-cerebral) may have a significant impact on the TIME, and could be a significant confounder given potential roles of the blood brain barrier. If these are available, the authors should perform similar immune analyses between samples obtained from intra-nasal vs. intra-cerebral tumors. Indeed, in Figure 1D, the authors find a decrease in number of CD4 helper cells in tumors with dural invasion, hinting that location of tumor sampling may be playing a role here.**

Intra-nasal vs intra-cerebral tumor location wasn’t taken into account when assembling the tissue microarray. The majority of ONB are intranasal and tumors with a large intra-cerebral component do not typically undergo surgical resection and oftentimes undergo induction chemotherapy first which likely alters the tumor immune microenvironment. Thus this question is difficult to directly assess due to limitations in tissue availability. We do have a recent treatment-naïve patient for which we collected tissue from both the intranasal and intracranial component for 10X 5’ GEX scRNA-Seq and TCR-Seq and is currently undergoing further analysis and is an area of further study.

**Minor comments:

1. For representative images shown in figures, please clarify whether these are from low or high grade tumors.**

The authors thank the reviewer for this helpful comment and have added high grade or low grade to the respective figure legend. **2. Throughout the figures, please make the labels on the IF images larger and more clear. Also please make the scale bars more visible on all images.**

Thanks for this helpful comment. We have enlarged the labels on IF images. Unfortunately, the scale bars are automatically added by the HALO® software and cannot be adjusted.

**3. In figure legends, please provide specifics on the statistical tests performed to calculate p-values shown in each figure.**

Thanks for this suggestion. The statistical test utilized has been added to each figure legend. **4. In Figure 4A, are these two tumors shown different Hyams grades?**

The authors would like to confirm that these two images are from different Hyams grade samples. The left column is a low Hyams grade sample (Hyams Grade I) and the column on the right is a high Hyams grade sample (Hyams grade III). The figure legend has been updated to clarify this point.

**5. The authors did not observe a correlation between MHC-I expression and the degree of CD8 T cell infiltration (line 266, Figure 4C). Interestingly, they did observe a correlation between HLA-DR+ tumor cell expression and the degree of CD4+ T helper cell tumor infiltration. As an internal control for any confounding effects, would the authors be able to comment on a correlation (or lack thereof) between 1) MHC-I expression and CD4+ T helper cells, and 2) HLA-DR+ tumor cells and CD8 T cell infiltration?**

Thanks for this question. No correlation was found between MHC-I^+^ tumor cells and CD4^+^ T cells (median of 30.96 cells per mm^2^ for low MHC-I expressing samples versus 8.03 cells per mm^2^ for high MHC-I expressing samples, p-value > 0.05). However, there was a correlation between HLA-DR^+^ tumor cells and CD8^+^ T cells (median of 2.05 cells per mm^2^ for low HLA-DR^+^ samples and 62.29 for high HLA-DR^+^ samples, p-value < 0.0001). Like CXCL9, CXCL10 and CXCL11 expression, HLA-DR expression on tumor cells is tightly linked to IFN-γ signaling. Given the very high constitutive expression of HLA class I observed in these tumors, HLA class II expression may act as a surrogate measure for the magnitude of T cell-derived IFN-γ in the local microenvironment. Given the close mechanistic association between IFN-γ signaling, more study is needed to understand the true mechanisms driving the heterogeneous HLA class II expression and T cell infiltration observed in these tumors. This has been added to the results and discussion sections please see lines 279-283 and 324-330 of the marked version of the manuscript.

**6. In line 325 of the discussion, findings is over-stated: when discussing their finding that Tregs are significantly closer in proximity to non-proliferating CD8+ cells compared to proliferating, the authors should tone down the language and not state that this "indicates their suppressive effect on the CD8+ T cell proliferation in ONB", since this is merely a correlational observation.**

Thanks for this comment. The verbiage has been adjusted to reflect this as a correlational observation. Please see lines 333-334 in the marked revised manuscript file.

**Reviewer #2: This is an excellent manuscript submitted by Dr. London and his team. ONB is a very rare tumor and hence the number of investigated samples is significant. Their findings will guide the design of further clinical trials.**

**They found significant T cell infiltration in olfactory neuroblastoma samples, especially within the stroma, and a significant decrease in MHC-I expression in high-grade olfactory neuroblastoma compared to low-grade disease. I think that this finding is particularly interesting and further work and wider collaborations will be able to demonstrate the mechanisms underlying this. Bearing in mind ongoing immunotherapy trials in this field, the findings described in their manuscript will be of particular interest for the readers and encourage other groups to use multispectral immunofluorescence and RNAScope evaluation of the tumor immune microenvironment in this rare tumor and other tumors.**

**The paper is well written and I would maybe only suggest to add at the end 'Advancements and emerging spatial transcriptomics approaches with formalin-fixed paraffin embedded tissue will allow for a more in-depth characterization at the single cell level in the future.' that ongoing multinational collaborations in the field of rare tumors will facilitate to help translate these findings into clinical practice.**

Thank you for these positive comments regarding the importance of our study. This statement was added to the manuscript as suggested. Please see lines 378-380 of the marked revised manuscript.
