# Supplementary material for: Augmentation of tumor expression of HLA-DR, CXCL9, and CXCL10 may improve olfactory neuroblastoma immunotherapeutic responses
Source: J Transl Med. 2024 May 31;22:524. doi: 10.1186/s12967-024-05339-9 (PMC11140921; doi:10.1186/s12967-024-05339-9)
Supplement: Supplementary file 4 — Supplementary Material 4 [file 12967_2024_5339_MOESM4_ESM.docx]

<http://www.google.com/url?sa=i&rct=j&q=&esrc=s&source=images&cd=&ved=2ahUKEwi36ISR9cjkAhUjp1kKHb7MD_gQjRx6BAgBEAQ&url=http%3A%2F%2Fphotography.jhu.edu%2Findex.php%2Fhopkins-logos%2F&psig=AOvVaw0MKx4jWopHxG78DJXclOsX&ust=1568296298478003>

___________________________________________________________________________

April 22, 2024

Dear Editor –

Please consider our revised manuscript JTRM-D-24-01200R1 entitled, “*Augmentation of tumor cell expression of HLA-DR, CXCL9, and CXCL10 may improve immunotherapeutic responses in olfactory neuroblastoma*” for publication as a Research Article. We have also included a point-by-point response addressing each of the reviewers’ comments.

Olfactory neuroblastoma is a very rare tumor (~300 cases per year in the United States), thus our collection of 47 clinically annotated tissue samples spanning the disease spectrum is a unique strength of our study and a highly valuable resource. This large population for such a rare tumor allowed us to ascertain many critical clinical correlations that have previously been unrecognized as well as provide exciting potential future paths for therapeutic targeting through mechanistic studies presented in this study. This first detailed analysis of the olfactory neuroblastoma tumor immune microenvironment is a landmark study for this field and a benchmark for future study of the tumor immune microenvironment for other rare tumors with wide reaching impact. Our group is the only team in the United States with an active immunotherapy clinical trial for olfactory neuroblastoma, thus we can use the results of this study to refine future immunotherapy clinical trial approaches.

We would like to point out that N. London receives grant funding from Merck to study HPV-related sinonasal malignancies which is not relevant to the present manuscript. All other authors declare no competing interests. The word count for this manuscript is 5,012 words.

Please do not hesitate to contact us with any questions.

Sincerely,

Nyall R. London Jr., M.D., Ph.D., F.A.C.S.
Johns Hopkins University School of Medicine

Associate Professor of Otolaryngology – Head and Neck Surgery

Principal Investigator – Sinonasal and Skull Base Tumor Program

National Cancer Institute (NCI)/National Institutes of Health (NIH)

+1-801-547-7851 (cell)
